# The Nuisance Mosquito *Anopheles plumbeus* (Stephens, 1828) in Germany—A Questionnaire Survey May Help Support Surveillance and Control

**DOI:** 10.3389/fpubh.2017.00278

**Published:** 2017-10-27

**Authors:** Eva C. Heym, Jette Schröder, Helge Kampen, Doreen Walther

**Affiliations:** ^1^Institute of Land Use Systems, Leibniz Centre for Agricultural Landscape Research, Muencheberg, Germany; ^2^Survey Design and Methodology, GESIS Leibniz Institute for the Social Sciences, Mannheim, Germany; ^3^Institute of Infectology, Friedrich-Loeffler-Institut, Federal Research Institute for Animal Health, Greifswald, Insel Riems, Germany

**Keywords:** *Anopheles plumbeus*, citizen science, mass development, mosquito control, tree-hole breeder, vector

## Abstract

The mosquito species *Anopheles plumbeus* is an aggressive biter and a potential vector of malaria parasites and West Nile virus. It occurs naturally at low population densities, as its larval development is adapted to the specific water qualities found in tree holes. However, probably owing to environmental changes, it has recently been observed in several European countries to use increasingly often artificial breeding habitats that may lead to mass development and severe annoyance to humans living close by. The perception of mosquito nuisance, however, is very subjective, and breeding habitats are not always known, thus impeding targeted surveillance and control. To relate nuisance by *An. plumbeus* to specific environmental conditions, a questionnaire survey was carried out addressing persons who had submitted specimens of this particular mosquito species to the German citizen science project “Mueckenatlas”, an instrument of passive mosquito surveillance. The questionnaire was intended to find out whether a nuisance situation linked to *An. plumbeus* had existed, whether mosquito breeding habitats could be identified and whether control measures had been conducted. Despite some efforts, the participants who claimed to suffer from an *An. plumbeus* nuisance problem had rarely identified the source of the mosquitoes. Once control measures had been performed on abandoned manure pits, however, the nuisance problem disappeared or mosquito abundance was at least significantly reduced. Nevertheless, no significant effect of abandoned manure pits on the probability of an *An. plumbeus* nuisance could be demonstrated in a multivariate logistic regression model testing various variables. Instead, a significant positive effect of a disused farm nearby was found. The reason is probably that manure pits as the most frequent source of *An. plumbeus* mass development are often located on disused farms, without most people’s knowledge about their existence. Disused farms are therefore appropriate candidates to consider when it comes to public health issues connected to *An. plumbeus* such as surveillance of mass development and implementation of control measures.

## Introduction

*Anopheles plumbeus*, a culicid mosquito widely distributed in Europe, has been identified as a major cause of severe mosquito nuisance in Germany (1). It is described as primarily dendrolimnic but has repeatedly been found breeding in artificial containers in recent decades (2–5). While tree holes can only produce small mosquito populations, mass development is often observed when artificial habitats are used as breeding sites (6, 7). Among these, abandoned manure pits are particularly critical, as they seem to offer ideal living conditions for enormous numbers of larvae (6). Mass development in connection with manure pits has been reported from Germany, the Netherlands, and Belgium (1, 7–9).

*Anopheles plumbeus* females feed indiscriminately on birds and humans, making them potential bridge vectors of zoonotic pathogens with avian reservoirs. Vector competence has been described for West Nile virus (WNV) and malaria parasites (10–12). WNV has been circulating in Europe for decades but has caused severe outbreaks among humans, horses, and birds only more recently (13). Infections in Germany have been diagnosed in migrating birds only, based on antibody detection (14). Autochthonous transmission of *Plasmodium falciparum* by *An. plumbeus* in Germany was suggested in 1997, when two cases of malaria occurred in persons without travel history (15). In addition, bites by *An. plumbeus* may result in fierce skin reactions and severe health problems, as humans are generally not accustomed to the bites of this mosquito species (6). Because it is both a potential vector and a potential pest, the surveillance of *An. plumbeus* outbreaks is of great public health importance.

While the incidence of diagnosed mosquito-borne disease cases in Europe has been negligible during recent decades and these have therefore been considered of minor importance, infections after mosquito bites, for example, with WNV, often remain undetected (16). The aggressive biting behavior and the bothersome buzzing noise of approaching mosquitoes on the other hand usually have an immediate negative impact on those affected. As a consequence, people are concerned with mosquitoes as pests rather than as vectors (17, 18). However, sensitivity toward annoyance by mosquitoes is very subjective, and in regions usually with low levels of mosquitoes residents feel more bothered by attacks than in regions where mosquitoes are numerous (19). In addition, problems caused by mosquitoes often occur on an unpredictable local scale, thus emphasizing the general public’s support in surveying them.

Mosquito control hardly exists in Germany. Only in the Southwest German Upper Rhine Valley have mosquitoes been routinely monitored over the last few decades and, if necessary, have been controlled to improve the life quality of people living along the floodplains of the River Rhine (20). In contrast to floodwater mosquitoes, which develop in masses only periodically after weather-related flooding of the meadows and can be controlled on a large scale, e.g., by releasing insecticides from a helicopter, *An. plumbeus* is a species that develops continuously and independently of weather events at clandestine sites and must be monitored and controlled manually according to those specific sites. Control usually consists of physical modifications of the environment such as elimination of artificial breeding habitats (1). While the latter might be laborious, the real challenge is to identify the sites of mass development.

In Germany, the community can contribute to mosquito surveillance and mapping via the citizen science project “Mueckenatlas” (Mosquito Atlas, www.mueckenatlas.de), which was launched in 2012 (21, 22). In this project, citizens may submit mosquitoes collected in their private surroundings for scientific analysis. They are asked to catch the mosquito in a closable container and put it into the freezer over night. This way, the mosquito is killed and remains intact for morphological identification (22). A special element of the project is that each participant will be contacted and will receive a feedback about which mosquito species has been collected.

In the framework of the “Mueckenatlas” project, *An. plumbeus* was frequently submitted by citizens claiming to suffer from a mosquito pest. To identify the ecological conditions at *An. plumbeus* sampling sites that might be related to this species becoming a pest, a questionnaire survey was designed using the experience of citizens dealing with this species. Specifically, the hypothesis that abandoned manure pits are positively correlated with *An. plumbeus* mass occurrence was tested. In addition, the identification and management of mosquito sources was requested. The results of the survey are designed to enhance readiness and to contribute to a more accurate and efficient surveillance of *An. plumbeus* mass development.

## Materials and Methods

### The Questionnaire

A written survey was carried out among all those who had submitted *An. plumbeus* specimens to the “Mueckenatlas” scheme between 2012 and 2015. This involved 150 persons living throughout Germany (1). The questionnaire contained a total of 20 questions related to mosquito nuisance, biological and behavioral characteristics of the submitted mosquitoes, potential mosquito breeding sites, control measures, and demographic data of the participants (Supplementary Material). For testing the intelligibility of the questionnaire, a preliminary test version had been distributed before the study to 20 persons with different education levels and without knowledge about the research topic. Only after their feedback, the final questionnaire version was prepared and distributed.

The major part of the questionnaire consisted of closed-ended questions where respondents were given a list of response choices from which they could select. In addition, the questionnaire contained some open-ended questions providing blank spaces where respondents were asked to draft an answer. Answers to open-ended questions were categorized before evaluation by summarizing identical descriptive answers.

Following questions about a potential mosquito nuisance situation, participants were asked whether they had potential breeding sites in their immediate environment, and if so, if they had identified those as actual breeding sites, as the identification of sources is decisive for control. Independently of that, they were asked if any control measures had been implemented, either directed against adult or aquatic mosquito stages, and, if so, of which kind they were.

Another question asked if there were contacts with the “Mueckenatlas” team beyond mere species identification (e.g., after receiving the identification result, some participants requested more detailed information on the submitted mosquito species). With this question, the possible influence of above-average knowledge about *An. plumbeus* mass development on successful management was examined.

### Data Analysis

To evaluate the knowledge about potential mosquito breeding sites and mosquito control efforts conducted by the participants, pairwise comparisons were conducted with the chi-square test or Fisher’s exact test using SPSS Statistics 22 (IBM).

For analyzing the determinants of nuisance by *An. plumbeus* using Stata 13 (StataCorp), logistic regression was applied (23). Since respondents were no mosquito experts, two indirect (dependent) variables were used. It was postulated that in the case of *An. plumbeus* mass occurrence, staying outdoors was highly restricted by the activity of the mosquitoes. Therefore, the participants were asked if the annoyance by mosquitoes affected their behavior and changed their outdoor activities. Respondents could answer in the affirmative or in the negative, so that a binary variable resulted, called “subjective nuisance”. The answer “yes” was regarded as an indication for the existence of a mosquito pest. As a mosquito pest, however, might not necessarily be caused by *An. plumbeus*, a further question asked if diurnal mosquito activity had been observed. In addition to the answer options “yes” and “no”, the option “do not know” was offered. Responses to the latter, however, were not included in the analysis.

Various characteristics of the surroundings of the mosquito collection site were considered as determinants of *An. plumbeus* mass occurrence. The main focus was put on the presence or absence of an abandoned manure pit nearby. Respondents were asked whether such a pit was known to exist within a diameter of about 500 m around their homes. The variable “abandoned manure pit nearby” was coded “1” if respondents agreed and “0” if the answer was “no” or “do not know”, thus producing a binary variable again.

Since people might not always be so familiar with their surroundings that they are aware of the existence of an abandoned manure pit, the variable “disused farm nearby” was used as an alternative indicator of the existence or absence of an abandoned manure pit. The answers “no” or “do not know” were again combined to one category.

Although natural breeding habitats are not expected to be linked to *An. plumbeus* mass occurrence, such an association was tested by two additional binary variables. The variable “forest nearby” captured the existence of a forest within a maximum distance of 500 m. The variable “green area nearby” combined respondents’ information on the existence of a park or a cemetery within the given distance. Both in green areas and in forests, trees with cavities can be expected to exist, offering natural breeding habitats for *An. plumbeus*.

In addition to these possible determinants of mass occurrence, several control variables were included in the multivariate regression: “farm nearby” measured the existence of a farm within a distance of 500 m and “rural environment” whether respondents classified their environment as rural.

Some respondent characteristics were also included in the regression analysis: the variable “age”, the dummy “high education” measuring whether the respondent’s highest graduation was at least “Abitur”, and the variable “gender”.

For both dependent variables, “subjective nuisance” and “diurnal mosquitoes”, three logistic regression models were run, all of them including the control variables as well as the determinants “forest nearby” and “green area nearby”. The first model also evaluated the variable “abandoned manure pit nearby”. The second model considered the variable “disused farm nearby” instead. The third model tested if an effect of the independent variables “abandoned manure pit nearby” and “disused farm nearby” on the dependent variables was still observed when both variables were analyzed at the same time. All models were estimated with robust standard errors using Huber–White sandwich estimators.

## Results

### Sociodemographic Characteristics of Survey Respondents

118 persons returned the questionnaire, resulting in a response rate of 78.6%. Table 1 gives an overview of the sociodemographic characteristics of the participants. According to the unbalanced “Mueckenatlas” submission rate of mosquitoes from the different German federal states, most of the respondents to the survey were from Bavaria and Baden-Wuerttemberg. Compared with the general population in Germany, older age groups, males and persons with a higher educational level (German “Abitur”) were overrepresented in this study. Most of the respondents pursued a job or were retired.

**Table 1:** Sociodemographic characteristics of survey participants.

Variable	Category	No. of respondents	%
Federal state	Baden-Wurttemberg	24	21.1
	Bavaria	25	21.9
	Berlin	2	1.8
	Brandenburg	14	12.3
	Hesse	6	5.3
	Mecklenburg-Western Pomerania	1	0.9
	Lower Saxony	7	6.1
	North Rhine-Westphalia	13	11.4
	Rhineland-Palatinate	4	3.5
	Saxony	12	10.5
	Saxony-Anhalt	5	4.4
	Thuringia	1	0.9
Age group (years)	0–19	3	2.8
	20–29	2	1.9
	30–39	13	12.0
	40–49	29	26.9
	50–59	26	24.1
	60–69	24	22.2
	70+	11	10.2
Gender	Male	76	67.9
	Female	36	32.1
Education	High school graduation (“Abitur”)	58	50.9
	Advanced technical college certificate	15	12.9
	Secondary school certificate	30	25.9
	Folk/secondary school or polytechnic secondary school	9	7.8
	Student/pupil	4	2.6
Occupation	Pupil	3	2.5
	Student	1	0.9
	Unemployed	1	0.9
	Housewife/househusband	10	8.7
	Pensioner	26	22.6
	Worker	70	60.9
	Other	4	3.5

### Knowledge about Mosquito Breeding Habitats

The majority of the people claimed to have no mosquito breeding habitats close to their homes or had no knowledge about them (46.0% of replies selected “existent”, 54.0% of replies “non-existent” or “unknown”; Table 2). Almost all respondents had a rain barrel and rain gutters in their immediate environment, which are suitable breeding sites for several mosquito species, but not for *An. plumbeus*. More than half of the respondents lived near a brook and knew that they had tree holes in their gardens. Only a small number of participants replied to the question as to whether these water sources had or had not been recognized as potential mosquito breeding habitats, whereas almost 60% of the respondents did not make an entry. 63.3% of those who replied had not identified these water reservoirs as mosquito sources.

**Table 2:** Identification of mosquito breeding habitats in the immediate environment of the respondents.

Potential breeding habitats in the environment of the respondents				Mosquito source identified^a^	
	
	Existent (%)	Non-existent (%)	Unknown (%)	Yes (%)	No (%)
Abandoned manure pit^b^	42 (38.2)	40 (36.4)	28 (25.5)	13 (31.0)	24 (57.1)
Active manure pit	41 (36.0)	48 (42.1)	25 (21.9)	8 (19.5)	22 (53.7)
Underground water reservoir^b^	28 (26.2)	41 (38.3)	38 (35.5)	5 (17.9)	17 (60.7)
Rain barrel	99 (86.1)	9 (7.8)	7 (6.1)	45 (45.5)	24 (24.2)
Well^b^	39 (35.5)	47 (42.7)	24 (21.8)	2 (5.1)	23 (59.0)
Rain gutter	94 (87.0)	9 (8.3)	5 (4.6)	5 (5.3)	52 (55.3)
Water pool, pond	74 (67.9)	30 (27.5)	5 (4.6)	22 (29.7)	18 (24.3)
Peat bog^b^	8 (7.7)	96 (92.3)	0 (0.0)	4 (50.0)	1 (12.5)
Brook	60 (54.5)	50 (45.5)	0 (0.0)	12 (20.0)	22 (36.7)
Ditch	26 (24.8)	72 (68.6)	7 (6.7)	5 (19.2)	10 (38.5)
Tree hole^b^	68 (61.8)	9 (8.2)	33 (30.0)	8 (11.6)	31 (44.9)
Other water sources^b^	26 (22.8)	84 (73.7)	4 (3.5)	14 (53.8)	3 (11.5)

*^a^The percentage is based only on positive answers (“existent”) given in “Potential breeding habitats in the environment of the respondents”*.

*^b^Breeding sites that are known to be used by Anopheles plumbeus*.

In general, respondents who had contacted the “Mueckenatlas” team for further information on the submitted mosquito species did not identify more potential breeding sites than those who had had no contact (*p* = 0.66). Despite this, there was a positive correlation between having had more intense contact with the “Mueckenatlas” team and knowledge about abandoned manure pits (*p* = 0.04).

Mosquito sources were not significantly more often identified by respondents who had reduced their outdoor activities due to the presence of mosquitoes (*p* = 0.65) as compared to respondents who did not feel restricted. Education level also had no significant effect on whether potential breeding sites were known or not (*p* = 0.95).

Breeding sites known to be associated with the development of *An. plumbeus*, such as manure pits, wells, or tree holes as the natural breeding habitats, were rarely identified in the immediate environment (Table 2).

### Mosquito Prevention and Control

**Figure 1:**
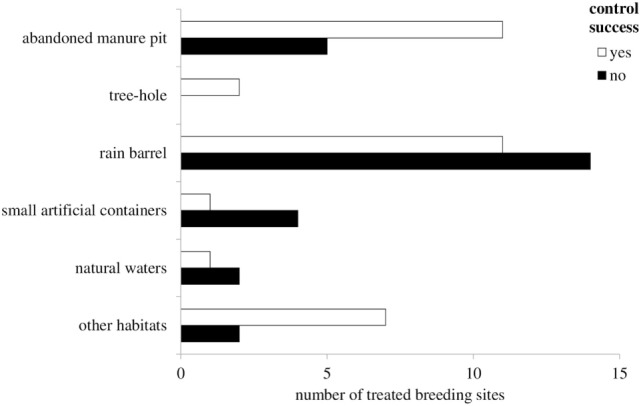
Outcome of mosquito control measures implemented for various types of breeding habitats.

A total of 56.8% (*n* = 67) of the study participants stated that they take action to tackle their mosquito problems. There was significantly more mosquito control when people felt constrained by mosquitoes (*p* < 0.001) and when mosquitoes were observed during daytime (*p* = 0.05). Of the respondents who had a rain barrel in their close surroundings and conducted mosquito control measures, control was predominantly targeted at the rain barrel (48.3%, *n* = 43). Information on the effect of the control measures was rare for all identified breeding sites, but trends were recognizable for rain barrels and manure pits. Control activities conducted on rain barrels were perceived as successful by 44% of the respondents (*n* = 11, Figure 1). In 68.8% (*n* = 11) of the cases where control measures had been conducted on manure pits or similar water sources, usually by drainage or filling with sand, people stated that the number of mosquitoes could be reduced (Figure 1). Mosquito control efforts were significantly more often directed at manure pits when the respondents had had more than usual contact with the “Mueckenatlas” team (*p* = 0.002). Only between two and nine replies about measures applied on other kinds of water sources were obtained.

### Relationship of *An. plumbeus* Mass Development and Abandoned Manure Pits

Altogether, 44% (*n* = 49) of the respondents stated that they were negatively affected by the activity of mosquitoes, coded as “subjective nuisance”, while 57% (*n* = 60) observed diurnal mosquitoes. Due to missing values on dependent and independent variables, 81 observations could be analyzed by the models with “subjective nuisance” as a dependent variable and 74 cases by the models with “diurnal mosquitoes” as a dependent variable. Descriptive statistics of all variables included in the multivariate analysis are shown in Table 3.

**Table 3:** Overview of the variables included in the regression analysis.

Variables	No. of observations	Percentage of positive answers
Subjective nuisance	81	51.6
Diurnal mosquitoes	74	55.4
Abandoned manure pit nearby	81	35.8
Disused farm nearby	81	59.3
Forest nearby	81	44.4
Green area nearby	81	44.4
Rural environment	81	84.0
Farm nearby	81	54.3
Gender (male)	81	66.6
Education level (“Abitur”)	81	51.9

	**Observations**	**Mean (SD)**

Age (min. 14–max. 80 years)	81	51.1 (14.4)

Table 4 displays the results for the three logistic regression models with the dependent variable “subjective nuisance” and the three models with the dependent variable “diurnal mosquitoes”. The focus of the models was on the effect of the independent variables “abandoned manure pit nearby” and “disused farm nearby” on the dependent variables. Results for model 1 do not show a significant effect of an abandoned manure pit nearby on “subjective nuisance” or on “diurnal mosquitoes” if the 5% significance level is applied.

**Table 4:** Logistic regression models analyzing the determinants of *Anopheles plumbeus* nuisance.

Tested variables	Subjective nuisance			Diurnal mosquitoes		
	Model 1	Model 2	Model 3	Model 1	Model 2	Model 3
	
	AME (*p*-value)	AME (*p*-value)	AME (*p*-value)	AME (*p*-value)	AME (*p*-value)	AME (*p*-value)
Abandoned manure pit nearby	0.22^+^ (0.09)	–	0.14 (0.31)	0.11 (0.42)	–	−0.02 (0.85)
Disused farm nearby	–	0.29* (0.04)	0.23 (0.13)	–	0.39** (0.00)	0.39** (0.00)
Forest nearby	−0.05 (0.66)	−0.04 (0.72)	−0.02 (0.89)	−0.12 (0.35)	−0.06 (0.63)	−0.06 (0.62)
Green area nearby	−0.11 (0.32)	−0.11 (0.30)	−0.09 (0.39)	−0.09 (0.45)	−0.07 (0.56)	−0.07 (0.55)
Rural environment	0.07 (0.65)	0.03 (0.87)	0.02 (0.92)	−0.09 (0.62)	−0.14 (0.43)	−0.13 (0.44)
Farm nearby	0.23^+^ (0.07)	0.19 (0.17)	0.16 (0.26)	0.11 (0.38)	−0.02 (0.91)	−0.01 (0.94)
Gender (male)	−0.23^+^ (0.05)	−0.20^+^ (0.09)	−0.20^+^ (0.08)	−0.21 (0.11)	−0.16 (0.22)	−0.16 (0.22)
Education level (“Abitur”)	−0.08 (0.45)	−0.07 (0.45)	−0.07 (0.47)	0.08 (0.47)	0.08 (0.45)	0.08 (0.45)
Age	−0.00 (0.27)	−0.00 (0.24)	−0.00 (0.26)	−0.00 (0.30)	−0.01 (0.21)	−0.01 (0.20)
*N*	81	81	81	74	74	74
Pseudo *R*^2^	0.21	0.22	0.23	0.12	0.19	0.19

^+^p < 0.10, *p < 0.05, **p < 0.01; p-values in parentheses.

*AME, average marginal effect*.

Model 2 shows a significant effect of a disused farm nearby on both dependent variables. The average marginal effect (AME) of a disused farm on nuisance is 0.29 (Table 4). This means that the estimated average probability (62%, not shown in Table 4) of having a nuisance problem when assuming that all of the respondents had a disused farm nearby is 29 percentage points higher than the estimated average probability (33%) of having a nuisance problem when assuming that none of the respondents had a disused farm nearby. The AME of a disused farm nearby on the probability of observing diurnal mosquitoes is 0.39 (probability if all had a disused farm nearby: 70%, probability if nobody had a disused farm nearby: 31%).

Model 3 includes both variables, “disused farm nearby” and “abandoned manure pit nearby”. The effect of “disused farm nearby” is smaller and not significant in the case of the dependent variable “subjective nuisance”, which means that part of the effect of the variable “disused farm nearby” on the variable “subjective nuisance” is mediated by the variable “abandoned manure pit nearby”.

According to the analysis, a green area or a forest located in the vicinity of the *An. plumbeus* collection site had no effect on the dependent variable. Apparently, nuisance does not appear to be associated with the natural breeding site of the species.

## Discussion

The major purpose of the survey was to check the linkage of an *An. plumbeus* nuisance situation with the existence of an abandoned manure pit nearby and the ability of people affected by such a nuisance to identify mosquito breeding sites and implement control measures. The effect of an abandoned manure pit was measured both directly and indirectly with the variable “disused farm nearby” as an indicator of the existence of an abandoned manure pit, since there was doubt that people knew for sure whether an abandoned manure pit existed in their neighborhood or not. Indeed, a significant effect of the reported existence of an abandoned manure pit nearby was neither found on the dependent variable “subjective nuisance” nor on “diurnal mosquitoes” the two measures of *An. plumbeus* mass development. By contrast, an effect of a disused farm nearby was demonstrated on both dependent variables when not testing for abandoned manure pits. This pattern is an indicator that abandoned manure pits do have an effect on *An. plumbeus* mass development although people may not know about them. Manure pits that are no longer used are often neglected or even forgotten (24). The fact that some people who had had problems with mass development and knew of an abandoned manure pit successfully conducted mosquito control on this pit adds to the missing significance of the effect of the variable “abandoned manure pit”. These results suggest that when monitoring *An. plumbeus* mass development it is not sufficient to ask people for abandoned manure pits but they should rather be asked whether a disused farm existed close by. An alternative explanation for the effect of disused farms on the occurrence of *An. plumbeus* is not plausible from the point of view of the biology of the mosquito.

As breeding habitats on private properties are the most important mosquito sources in settlements, the advice offered by residents about potential breeding sites is essential for successful mosquito control. In this study, only a few potential mosquito breeding habitats were identified by the participants, even if these were present on their private premises. Whether these potential breeding sites were or were not actual mosquito sources could not be clarified in the scope of this study. A limited awareness of artificial containers as mosquito breeding sites was also shown in a survey from western Australia, despite mosquito-borne diseases affecting one billion people in this region annually (25).

The acceptance of abandoned manure pits or similar organic-rich stagnant underground water reservoirs as breeding sites for *An. plumbeus* is known only to a few people. Respondents to the survey stated that the number of mosquitoes was in most cases successfully reduced following implementation of control efforts on manure pits. If control was not successful, this might be because only the complete elimination of the habitat can guarantee a sustainable reduction in mosquito abundance (1). Information about abandoned manure pits by the “Mueckenatlas” team contributed to a greater probability of source identification, which underlines the importance of the cooperation between experts and the general public. Generally, citizens prefer to control mosquito populations on their own, i.e., without the help of official public health bodies, even in West Nile fever risk areas (26). On the one hand, this is because mosquito-borne diseases are not considered a risk, but on the other hand because of limited faith in public pest control operators and a refusal to allow the spraying of adulticides (26). In fact, mosquito adulticide spraying usually has a limited effect, as populations quickly recover (27).

A study in Tanzania, where malaria constitutes a major public health problem, has shown that even if residents were aware of the link between their problems and mosquitoes, attempts to reduce or eliminate mosquito breeding places were often inefficient, as little information about the differing importance of different kinds of water sources was distributed (28).

In Colombia, dengue and *Aedes aegypti* mosquitoes have become routine business, but public health measures are rather directed against consequences than against causes and are mainly based on misinformation. For instance, fumigation of adult mosquitoes is seen as paramount by the citizens whereas breeding-site management is neglected, the more so as the commonly known breeding sites, car tires, are confined to industrial areas (29). Indeed, another study from Colombia, carried out in 2014, has shown that if control measures are directed against the mass breeding sites of *Ae. aegypti*, a significant decrease of the *Aedes* population and, consequently, of dengue can be observed (30).

That the activity of affected residents can be particularly effective in reducing the infection risk posed by mosquitoes was demonstrated by a survey conducted in a West Nile fever risk area in Colorado, USA in 2003 (31): Persons living in a city with higher mosquito-biting pressure were more likely to take preventive measures and were therefore better protected against mosquito bites than persons living in a city with lower biting activity.

In summary, it appears to be appropriate to ask residents about disused farms in addition to abandoned manure pits when trying to identify sources of *An. plumbeus* mass development. As most of the respondents to the present study had not identified the *An. plumbeus* breeding sites, more education is necessary for the general public to recognize potential mosquito breeding habitats and to prevent artificial water reservoirs from becoming sources of mosquito mass development.

### Study Limitations

The main limitation of this study is that the survey was restricted to people who submitted *An. plumbeus* to the “Mueckenatlas”. It would be desirable of course to have a survey of randomly sampled people living in Germany.

As only participants to the citizen science project “Mueckenatlas” were included in the study, the survey did not consider all social levels and age groups (e.g., retired people spend more time at home and have a higher likelihood of registering diurnal mosquitoes than active workers).

Furthermore, some parts of Germany were better represented by submissions than other parts, which could be due to the distribution of the species, but also caused by the regionally different media attention of the “Mueckenatlas”. In addition, it could not be verified if water sources present but not identified by the respondents were mosquito breeding habitats or not.

## Ethics Statement

All procedures were performed in accordance with international and German law, with written informed consent by all participants. All given information was evaluated anonymously.

## Author Contributions

EH and DW: conceived and designed the study. DW and HK: provided mosquito data. EH and JS: analyzed the data. EH, JS, HK, and DW: wrote the paper.

## Conflict of Interest Statement

The authors declare that the research was conducted in the absence of any commercial or financial relationships that could be construed as a potential conflict of interest. The reviewer VT and handling editor declared their shared affiliation.

## Supplementary Material

Questionnaire, translated into English for the purpose of this contribution. As the questionnaire was addressed to German citizens it had been distributed in German language.
