# Supplementary material for: The Nuisance Mosquito Anopheles plumbeus (Stephens, 1828) in Germany—A Questionnaire Survey May Help Support Surveillance and Control
Source: Front Public Health. 2017 Oct 27;5:278. doi: 10.3389/fpubh.2017.00278 (PMC5663692; doi:10.3389/fpubh.2017.00278)
Supplement: Supplementary file 1 [file Data_Sheet_1.PDF]

MUECKENATLAS - QUESTIONNAIRE

1. How did the Mueckenatlas project come to your attention? (please tick one answer only)

- ☐ TV
- ☐ broadcast
- ☐ newspaper
- ☐ professional organisation
- ☐ friends
- ☐ internet
- ☐ other (please specify:____)

2. Have you ever contributed to a community-based scientific project before?

ja ☐

nein ☐

2a. If so, which?____

3. Did you contribute to the Mueckenatlas project repeatedly?

yes, several times ☐

no, only once ☐

4. What did you make contributing to the Mueckenatlas scheme when you submitted first?
(please tick where appropriate; multiple answers possible)

- ☐ you wanted to know about the mosquito species
- ☐ you wanted to support science and research
- ☐ you wanted to gather education (generally)
- ☐ recommendation by acquaintances/friends
- ☐ interest in your own garden/home environment
- ☐ general interest in nature
- ☐ other (please specify: ____)

5. Which of the following applies regarding the surroundings of the collection site of your first Mueckenatlas mosquito submission? (please tick in every line)

	applies	does not apply	do not know
predominantly rural environment	☐	☐	☐
predominantly urban environment	☐	☐	☐
collected in my garden	☐	☐	☐
collected in my apartment/house	☐	☐	☐
farm nearby (distance up to 500m)	☐	☐	☐
manure pit nearby (distance up to 500m)	☐	☐	☐
cemetery nearby (distance up to 500m)	☐	☐	☐
park nearby (distance up to 500m)	☐	☐	☐
forested area nearby (distance up to 500m)	☐	☐	☐
floodplains nearby (distance up to 500m)	☐	☐	☐
cannot remember	☐	☐	☐
other (please specify:_____________________ _______________________________________)	☐	☐	☐

6. In which situation did you encounter the submitted mosquito? (please tick where appropriate)

- ☐ approaching/biting humans
- ☐ approaching/biting animals
- ☐ found dead (indoors e.g., on the window sill, or outdoors)
- ☐ flying around/resting outdoors
- ☐ resting indoors (cellar, room wall, loft)
- ☐ other (please specify:_________________________________)

7. Did you notice peculiarities of the first mosquito submitted? If so, which: (please tick where appropriate)

- ☐ color (please specify: __________________________________)
- ☐ extraordinarily aggressive
- ☐ particularly large
- ☐ particularly small
- ☐ extraordinarily abundant
- ☐ other (please specify:___________________________)
- ☐ no peculiarities

8. Where in your neighborhood are you attacked/bitten most by mosquitoes?

(please tick where appropriate)

- ☐ in my apartment/house
- ☐ in my garden
- ☐ equally often in my apartment/house and my garden
- ☐ during walks in the neighborhood
- ☐ other (please specify:______________________________)

9. Should your first submission have contained several mosquitoes, did you have the feeling that they belonged to different or the same species?

- ☐ different species
- ☐ one single species only
- ☐ I submitted only one single specimen

10. Nuisance by mosquitoes can vary and depends on personal perception. Please rate the following statements. (please tick where appropriate)

	applies	does not apply	do not know
I rarely spot a mosquito at my home	☐	☐	☐
Mosquitoes become annoying at my home only for a few days in summer.	☐	☐	☐
Mosquitoes become annoying only at dusk.	☐	☐	☐
During summer, mosquitoes are active and attack all day in my home/garden/ neighborhood	☐	☐	☐
Protection from mosquitoes (e.g. repelling spray) is necessary to enjoy outdoor activities during summer in my garden/the neighborhood.	☐	☐	☐
When no measures are taken, e.g. closing the windows, mosquitoes also enter the apartment/house and attack indoors.	☐	☐	☐
Caused by mosquito activity, leisure time can hardly be spent in my garden/neighborhood.	☐	☐	☐
Owing to significant nuisance by mosquitoes, friends have become reluctant to visit our house.	☐	☐	☐
Owing to the continuous nuisance by mosquitoes we consider moving away.	☐	☐	☐
During the recent years, nuisance by mosquitoes has increased.	☐	☐	☐
other (please specify: ________________________ ____)	☐		

11. Does annoyance by mosquitoes at your home/in your neighborhood influence your behavior, e.g. leisure activities?

yes (please continue with question 12) ☐
no (please continue with question 17) ☐

12. When did the annoyance by mosquitoes start to influence your behavior?
(please specify year if known) ________________________________

13. Please try to rate the degree of annoyance:
(tick box: 1 - negligible; 10 - extreme, no outdoor activities possible)

negligible extreme

☐	☐	☐	☐	☐	☐	☐	☐	☐	☐
1	2	3	4	5	6	7	8	9	10

14. Please rate the annoyance during the past few years (tick one box per year):

	negligible	medium	strong
2011	☐	☐	☐
2012	☐	☐	☐
2013	☐	☐	☐
2014	☐	☐	☐
2015	☐	☐	☐

15. Please try to rate the annoyance during the course of the year:
(tick box: X – negligible/no annoyance, XX – medium, XXX – strong)

☐	☐	☐	☐	☐	☐	☐	☐	☐	☐	☐	☐
Jan	Feb	Mar	Apr	May	Jun	Jul	Aug	Sep	Oct	Nov	Dec

16. At about what time of the day are the mosquitoes most active during the months of highest nuisance? (please tick time)

☐	☐	☐	☐	☐	☐	☐	☐	☐	☐	☐	☐	☐	☐	☐	☐	☐	☐	☐	☐	☐	☐	☐	☐	
1	2	3	4	5	6	7	8	9	10	11	12	13	14	15	16	17	18	19	20	21	22	23	24	o'clock

17. Are there abandoned farms or agricultural production sites close to your home?

yes ☐
no ☐
not known ☐

17a. If yes, how far away (approximately)?

- ☐ 0 - 100m
- ☐ 100 - 200m
- ☐ 200 - 300m
- ☐ 300 - 400m
- ☐ 400 – 600m
- ☐ more than 600m

18. Please tick in the following list of possible mosquito breeding habitats whether the specific habitat can be found in your neighborhood and, if so, whether this has been identified as a possible mosquito source.

	existent			identified as mosquito source	
	yes	no	do not know	yes	no
abandoned manure pit	☐	☐	☐	☐	☐
used manure pit	☐	☐	☐	☐	☐
subterranean water reservoir	☐	☐	☐	☐	☐
rain water barrel	☐	☐	☐	☐	☐
well	☐	☐	☐	☐	☐
rain gutter	☐	☐	☐	☐	☐
pool	☐	☐	☐	☐	☐
lake	☐	☐	☐	☐	☐
bog	☐	☐	☐	☐	☐
creek/river bank	☐	☐	☐	☐	☐
ditch	☐	☐	☐	☐	☐
tree-holes	☐	☐	☐	☐	☐
other water sources (please specify:_________________)	☐	☐	☐	☐	☐

19. Did you take to mosquito control actions in your home/garden (e.g., application of chemicals, physically by covering rain water barrels or similar)?

yes ☐

no (continue with question 20) ☐

19a. If yes, please specify:

19b. After taking action, did the mosquito situation change to the better?

yes ☐
no ☐

20. In addition to mere submitting a mosquito/mosquitoes to the Mueckenatlas scheme and receiving a response, did you have further contacts to the Mueckenatlas team?

yes ☐
no ☐

21. For statistical purposes, please give some information on your personal background.

In which year were you born? ____________________________

What applies best to your person? (tick one box only)

- ☐ pupil
- ☐ unemployed
- ☐ student
- ☐ house wife/house husband
- ☐ retired
- ☐ employed (fully/part-time)
- ☐ other (please specify: _______________________________)

Which is your highest school graduation?

- ☐ still pupil
- ☐ no school graduation
- ☐ folk/secondary school or polytechnic secondary school
- ☐ secondary school certification
- ☐ advanced technical college certification
- ☐ high school graduation (German Abitur)

How many persons live in your household? _____________________

Do you have children who contributed to the Mueckenatlas scheme?

yes ☐
no ☐

(If yes, please specify the age of the children: __________________)
